# The role of cGAMP via the STING pathway in modulating germinal center responses and CD4 T cell differentiation

**DOI:** 10.3389/fimmu.2024.1340001

**Published:** 2024-04-12

**Authors:** Mijung Yoon, Yurim Choi, Taeuk Wi, Youn Soo Choi, Jinyong Choi

**Affiliations:** ^1^ Department of Microbiology, Department of Biomedicine & Health Sciences, College of Medicine, The Catholic University of Korea, Seoul, Republic of Korea; ^2^ Department of Biomedical Sciences, Department of Medicine, Seoul National University College of Medicine, Seoul, Republic of Korea; ^3^ Transplantation Research Institute, Seoul National University Hospital, Seoul, Republic of Korea

**Keywords:** STING ligand, cGAMP, germinal center, follicular helper T cells, plasma cells

## Abstract

Germinal center (GC) responses are essential for establishing protective, long-lasting immunity through the differentiation of GC B cells (B_GC_) and plasma cells (B_PC_), along with the generation of antigen-specific antibodies. Among the various pathways influencing immune responses, the STING (Stimulator of Interferon Genes) pathway has emerged as significant, especially in innate immunity, and extends its influence to adaptive responses. In this study, we examined how the STING ligand cGAMP can modulate these key elements of the adaptive immune response, particularly in enhancing GC reactions and the differentiation of B_GC_, B_PC_, and follicular helper T cells (T_FH_). Employing *in vivo* models, we evaluated various antigens and the administration of cGAMP in Alum adjuvant, investigating the differentiation of B_GC_, B_PC_, and T_FH_ cells, along with the production of antigen-specific antibodies. cGAMP enhances the differentiation of B_GC_ and B_PC_, leading to increased antigen-specific antibody production. This effect is shown to be type I Interferon-dependent, with a substantial reduction in B_PC_ frequency upon interferon (IFN)-β blockade. Additionally, cGAMP’s influence on T_FH_ differentiation varies over time, which may be critical for refining vaccine strategies. The findings elucidate a complex, antigen-specific influence of cGAMP on T and B cell responses, providing insights that could optimize vaccine efficacy.

## Introduction

The orchestration of adaptive immunity involves a complex interplay between various cells and molecular signals that ensure effective protection against pathogens. Germinal center (GC) response, including the differentiation of GC B cells (B_GC_) and plasma cells (B_PC_), and subsequent the production of antigen-specific antibodies, is critical for protective and long-term immunity (1, 2). The germinal center reaction is a pivotal process in the adaptive immune response, leading to the selection of B cells with high-affinity receptors. B_GC_ cells then differentiate into plasma cells or memory B cells, which are essential for producing high-affinity antibodies and conferring long-lasting immunity (1, 3, 4). Follicular helper T cell (T_FH_), a specialized B cell help CD4 T cell subset, is indispensable for the GC reaction, supporting the differentiation of B_GC_ cells and the affinity maturation (5–7).

The STING (Stimulator of Interferon Genes) pathway has emerged as a key player in innate immunity, influencing the adaptive immune branches as well (8). Recent studies have highlighted the significance of STING ligands, such as cyclic GMP-AMP (cGAMP) and its derivatives, in enhancing protective immunity against a range of pathogens, including SARS-CoV-2 and influenza viruses (9–12). These ligands have been shown to potentiate immune responses, yet their role in modulating the germinal center reaction remains to be fully elucidated. The specific impacts of STING activation on the differentiation of B_GC_ cells and plasma cells, as well as the kinetics and quality of antigen-specific antibody responses, need further exploration. Moreover, the ability of cGAMP to modulate T_FH_ cells poses intriguing questions about the broader implications of STING activation in vaccine and adjuvant designs.

In the present study, we aimed to dissect the influence of cGAMP on these critical components of the adaptive immune response. By employing various antigens and analyzing the differentiation and response kinetics of B_GC_, B_PC_, and T_FH_ cells, and the production of antigen-specific antibodies *in vivo* models, we explored to elucidate the mechanisms by which STING ligands can enhance the efficacy of immune responses. Understanding these mechanisms is imperative for designing effective vaccine adjuvants and therapeutic strategies in relevant biomedical fields, including cancer vaccines.

## Materials and methods

### Mice

C57BL/6J mice were acquired from Orient Bio in Korea. CD45.1^+^ SMARTA mice were kindly provided by Youn Soo Choi at Seoul National University College of Medicine. Specific-pathogen-free male mice (6-8 weeks of age) were used for experiments. All procedures involving animals were conducted in compliance with the protocols approved by the Institutional Animal Care and Use Committee of the College of Medicine at The Catholic University of Korea (CUMC-2023-0164).

### Adoptive cell transfer

Spleens from SMARTA mice were harvested, and naive CD4^+^ T cells were isolated using negative selection according to the manufacturer’s protocol (The EasySep™ Mouse CD4^+^ T Cell Isolation Kit, STEMCELL, #19852). The cells were counted, and the proportions of TCRVα2^+^ CD45.1^+^ CD4^+^ T cells were determined by flow cytometry. 5 x 10^4^ (for day 8) or 10 x 10^4^ (for day 15) naive SMARTA T cells (CD45.1^+^) were adoptively transferred into recipient mice (CD45.2^+^) via intravenous injection into the retroorbital sinus.

### Protein immunization

For immunization, 5 μg of SARS-CoV-2 spike RBD (sRBD), 10 μg of KLH-NP, or 10 μg of KLH-gp_61_ were each mixed with Alum (Alhydrogel; Invivogen) alone or combined with 5 μg of 3′3′-cGAMP (Invivogen) to a final volume of 20 μL. These preparations were administered into each footpad of the mice. The chosen cGAMP concentration was based on its ability to induce B_GC_ and B_PC_ responses in dose-response trials using the KLH-NP antigen (1, 5, and 10 μg). A 5 μg cGAMP dosage reliably provoked B_GC_ and B_PC_ responses, with 10 μg showing no further impact. For IFN-β blockade, mice received 250 μg of anti-IFN-β blocking antibody or isotype control via intraperitoneal injection three times during the course of protein immunization.

### Flow cytometry

Single-cell suspensions from popliteal or inguinal lymph nodes were stained with a panel of monoclonal antibodies for surface markers including CD4 (RM4-5, BV510), CD8 (53-6.7, BV605), SLAM1 (TC15-12F12.2, PepCP-Cy5.5), ICOS (C398.4A, FITC), IgD (11-26c.2a, PE), CD138 (281-2, APC), Streptavidin (BV421) (BioLegend), B220 (RA3-6B2, AF700), CD44 (IM7, PE.Cy7), PD1 (J43, PE), CD4 (RM4.5, APC-eF780), CD8 (53-6.7, APC-eF780), CD45.1 (A20, APC-eF780 or BV605), Fixable Viability Dye eF780 (eBioscience) and FAS (Jo2, BV510) (BD Biosciences) at 4°C for 30 min in PBS with 0.5% BSA.

For cytokine detection, the cells were *in vitro* cultured with 10 μg/mL gp_66-77_ and 1 μg/mL brefeldin A for 5 hours. Intracellular staining for cytokines was performed with monoclonal antibodies for IL-4 (11B11, PE), IFN-γ (XMG1.2, AF700) (eBioscience), IL-2 (JES6-5H4, PE.Cy7) (BioLegend), and recombinant mouse IL-21 receptor Fc (R&D systems), followed by anti-human IgG (DyLight650) (Invitrogen). A Fixation/Permeabilization kit (BD Biosciences) was employed for permeabilization. Intracellular staining for T-bet transcription factor was performed with monoclonal antibody T-bet (4B10; PerCP.Cy5.5) using the Foxp3/Transcription Factor Staining Buffer Set (eBioscience). The cells were then analyzed using an LSR Fortessa (BD Biosciences) and FlowJo software v.10.8.

### ELISA

Flat-bottom immuno plates (MaxiSorp) were coated with 1 μg/mL of various antigens (sRBD, BSA-gp_61_, OVAL-NP_(17)_, BSA-NP_(2)_ for high-affinity antibodies, and BSA-NP_(36)_ for low-affinity antibodies) in PBS at 4°C overnight. Plates were washed with PBST (PBS + 0.1% Tween-20) and blocked with PBST-B (PBS with 0.05% Tween-20 and 0.5% BSA) for 90 min at room temperature. Mouse sera were then added in serial dilution and incubated for 90 min at room temperature. After washing, HRP-conjugated goat anti-mouse IgG (Thermo Fisher Scientific) was added in PBST-B (1:5,000), and the plates were incubated for another 90 min at room temperature. The enzymatic reaction was developed with a TMB solution (BioLegend). The reactions were stopped with 2N sulfuric acid, and the absorbance was measured at 450 nm with a microplate reader.

## Results

### cGAMP enhances the differentiation of germinal center B cells, plasma cells, and antigen-specific antibody production

**Figure 1:**
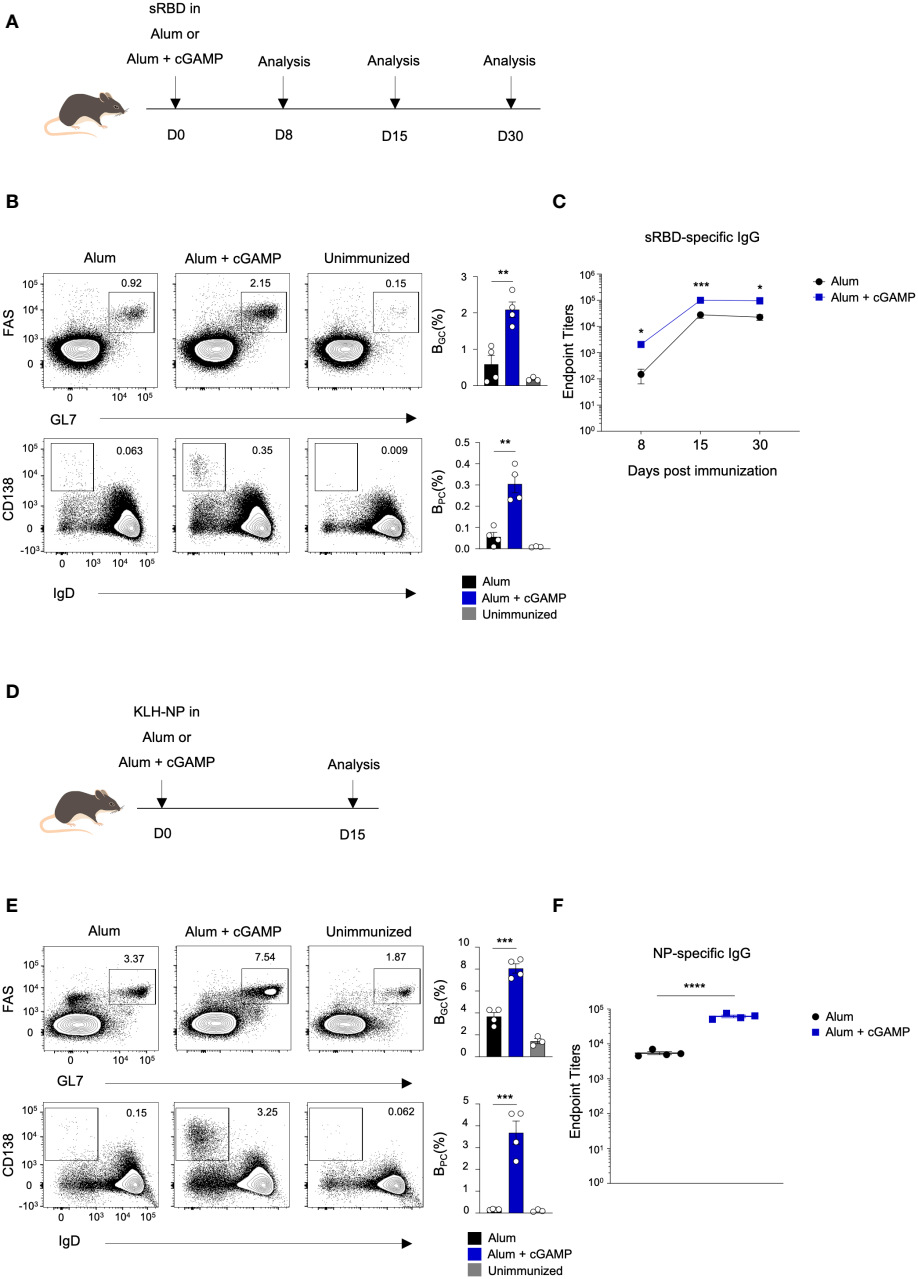
cGAMP enhances differentiation of B_GC_, B_PC_, and antibody production in response to sRBD and KLH-NP antigens. **(A)** Mice were immunized with sRBD in Alum or Alum+cGAMP. Draining lymph nodes (LNs) and blood were collected at 8, 15, and 30 days post-immunization (n=4). **(B)** Flow cytometry plots show the percentages of B_GC_ (GL7^hi^FAS^hi^) and B_PC_ (IgD^lo^CD138^hi^) on day 8. **(C)** sRBD-specific IgG endpoint titers were measured at 8, 15, and 30 days post-immunization. **(D)** Mice were immunized KLH-NP in Alum or Alum+cGAMP and were analyzed on day 15 (n=4). **(E)** Flow cytometry plots show the percentages of B_GC_ and B_PC_ on day 15. **(F)** NP-specific IgG endpoint titers were measured by ELISA. All experiments were performed twice, and each dot represents one mouse. Data are shown as mean ± SEM and were analyzed using an unpaired two-tailed Student’s t-test. *p < 0.05, **p < 0.01, ***p < 0.001, ****p < 0.0001.

STING ligands, such as 2’3’-cGAMP, 3’3’-cGAMP, and their derivatives, have been shown to enhance protective immunity against various pathogens, including SARS-CoV-2 and influenza (9–11). However, the impact of STING ligands on the germinal center reaction has remained somewhat unclear. To investigate the effect of STING ligands on the differentiation of B_GC_, B_PC_, and the generation of antigen-specific antibodies, we conducted experiments employing 3’3’-cGAMP (cGAMP) in response to various antigens. Mice were immunized with the SARS-CoV-2 spike RBD protein (sRBD) as an antigen, either in Alum alone or Alum+cGAMP as an adjuvant (**Figure 1A**). Mice immunized with Alum+cGAMP adjuvant exhibited a significant increase in frequencies of B_GC_ (Fas^hi^GL7^hi^) cells and B_PC_ (IgD^lo^CD138^hi^) compared to the mice immunized with Alum alone on day 8 after immunization (**Figure 1B**). The kinetics of B_GC_ and B_PC_ differed significantly. The increase in B_GC_ frequency induced by Alum+cGAMP persisted for 30 days with the peak response on day 15 after immunization. In contrast, the variance in B_PC_ frequency peaked on day 8 and gradually declined over one month post-immunization (**Supplementary Figure 1A**). Consistent with a previous report (9), sRBD-specific antibody titers were significantly higher in mice treated with Alum+cGAMP compared to those treated with Alum alone (**Figure 1C**).

We further investigated whether the effect of cGAMP on B_GC_ and B_PC_ is a general process in immunization with different antigens. Mice were immunized with keyhole limpet hemocyanin (KLH) conjugated with hapten 4-hydroxy-3-nitrophenyl acetyl (NP) (KLH–NP) as a model antigen, either in Alum alone or Alum+cGAMP (**Figure 1D**). Similarly, B_GC_ and B_PC_ responses were enhanced in mice treated with Alum+cGAMP in response to KLH–NP immunization (**Figure 1E**), along with an increase in NP-specific antibody production (**Figure 1F**). The increased frequency of both B_GC_ and B_PC_ was sustained for over one month when mice were immunized with Alum+cGAMP for priming (day0) and boosting (day14), suggesting that cGAMP treatment in primary and boost immunization can prolong the GC response and the differentiation of B_PC_ (**Supplementary Figures 1B,C**). Moreover, the magnitude of high-affinity (NP_(2)_; NP_2_-bound) antibody production was significantly greater in the Alum+cGAMP group compared to the control Alum alone group (**Supplementary Figure 1D**). These data demonstrate that cGAMP enhances the differentiation of GC B cells, plasma cells, and the production of antigen-specific antibodies.

### Enhancement of plasma cell differentiation by cGAMP is type I Interferon-dependent

**Figure 2:**
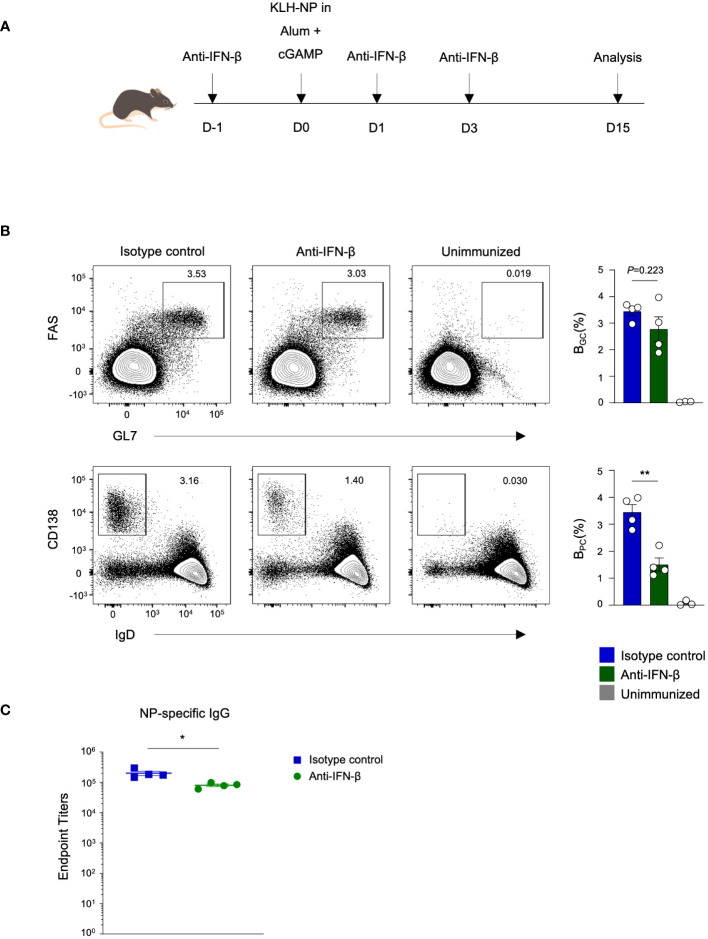
cGAMP augments antigen-specific antibody production and B_PC_ differentiation via a type I interferon-dependent mechanism. **(A)** Mice were immunized with KLH-NP in Alum or Alum+cGAMP and received 250 μg of anti-IFN-β blocking antibody or isotype control treatments three times. Analyses were conducted on day 15 (n=4). **(B)** Flow cytometry plots show the percentages of B_GC_ and B_PC_ on day 15. **(C)** NP-specific IgG endpoint titers were measured by ELISA. All experiments were performed twice, and each dot represents one mouse. Data are shown as mean ± SEM and were analyzed by using an unpaired two-tailed Student’s t-test. *p < 0.05, **p < 0.01.

Ligation of cGAMP to STING triggers the production of type I interferon (IFN) (13). To explore whether cGAMP has the potential to enhance the germinal center response through the type I interferon pathway, we immunized mice with KLH–NP in Alum+cGAMP and administered either anti-IFN-β blocking antibodies or isotype control antibodies before (D-1) and after (D1 and D3) immunization (**Figure 2A**). Interestingly, there were no significant changes in the frequency of B_GC_ in anti-IFN-β group. However, the frequency of B_PC_ was significantly reduced upon blocking IFN-β (**Figure 2B**). Consistent with the B_PC_ response, the production of NP-specific IgG was also decreased (**Figure 2C**). In summary, cGAMP plays a critical role in enhancing the differentiation of germinal center B cells and plasma cells, ultimately resulting in the production of antigen-specific antibodies. Specifically, cGAMP regulates plasma cell generation through the type I interferon pathway.

### cGAMP differently impacts T_FH_ differentiation in response to various antigens

**Figure 3:**
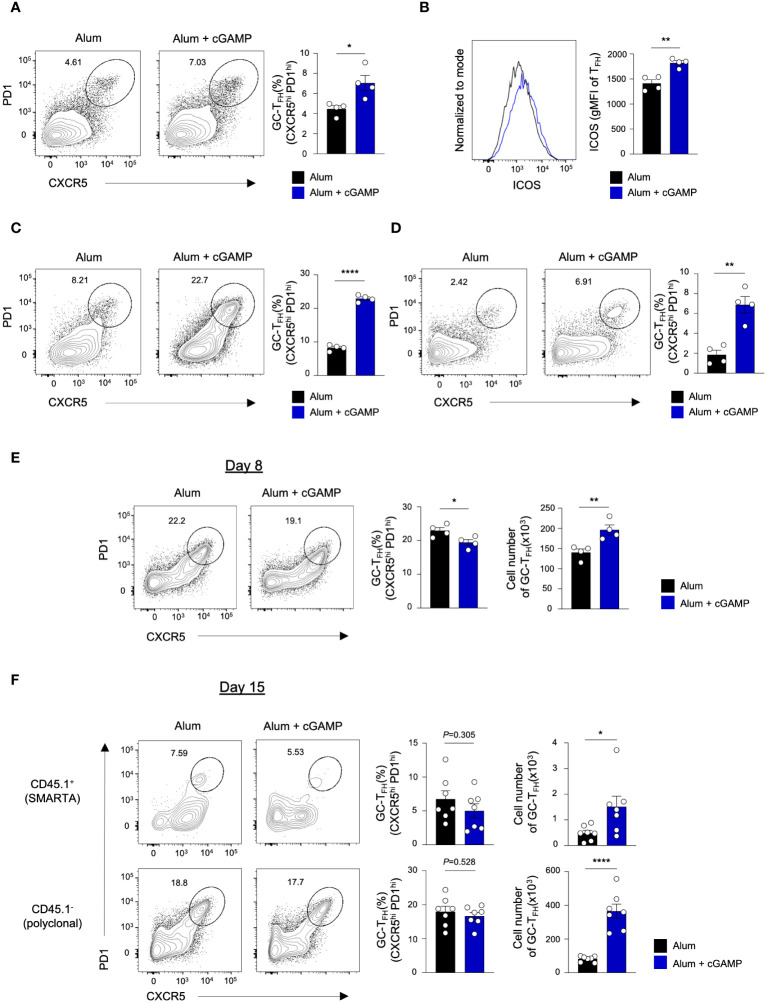
cGAMP promotes differentiation of GC-T_FH_ in response to various antigens. **(A, B)** Mice were immunized with sRBD in Alum alone or Alum+cGAMP. GC-T_FH_ population and ICOS expression levels on the CXCR5^+^ T_FH_ population were analyzed by flow cytometry on day 15 (n=4). **(C)** Mice were immunized with KLH-NP in Alum alone or Alum+cGAMP. GC-T_FH_ population was analyzed by flow cytometry on day 15 (n=4). **(D)** Mice underwent priming on day 0 and a booster on day 14 with KLH-NP in Alum alone or Alum+cGAMP. GC-T_FH_ population was analyzed by flow cytometry 20 days after the booster immunization (day 34, n=4). **(E)** Mice were immunized with KLH-gp_61_ in Alum alone or Alum+cGAMP. GC-T_FH_ population was analyzed by flow cytometry on day 8 (n=4). **(F)** SMARTA CD4^+^ T cells were adoptively transferred to mice, followed by KLH-gp_61_ immunization in Alum or Alum+cGAMP. Frequencies and cell number of GC-T_FH_ in endogenous polyclonal CD4 T cells (CD45.1^-^) and adoptively transferred SMARTA cells (CD45.1^+^) were analyzed by flow cytometry on day 15 (n=7). All experiments were performed twice, and each dot represents one mouse. Data are shown as mean ± SEM and were analyzed by using an unpaired two-tailed Student’s t-test. *p < 0.05, **p < 0.01, ****p < 0.0001.

T_FH_, a subset of CD4 T cells, plays a crucial role in GC formation, B cell differentiation and affinity maturation, and the production of high-affinity antibodies by producing plasma cells (1). We explored the potential of cGAMP to promote T_FH_ differentiation. Notably, cGAMP significantly enhanced the frequency of GC T_FH_ (CXCR5^hi^PD1^hi^) following sRBD immunization (**Figures 1A**, **3A**). Furthermore, cGAMP markedly upregulated the expression of inducible T-cell costimulator (ICOS), a critical costimulatory molecule for T_FH_ differentiation and synapse formation with B cells (**Figure 3B**). This upregulation was also observed in mice immunized with KLH-NP antigen, where cGAMP increased the frequency of GC T_FH_ cells compared to immunization with Alum alone (**Figures 1D** , **3C**). Importantly, the presence of GC T_FH_ cells was sustained for over a month when mice received prime-boost immunization with KLH-NP in Alum+cGAMP, unlike those immunized with Alum alone (**Figure 3D** and **Supplementary Figure 1B**), indicating the capacity of cGAMP to induce T_FH_ differentiation.

The effect of cGAMP on T_FH_ response to sRBD and KLH-NP antigens corresponds with the enhanced formation of B_GC_ and B_PC_. Intriguingly, despite cGAMP’s known role in promoting T_H_1 differentiation and IFN-γ production through STING activation in CD4 T cells, it can also augment T_FH_ differentiation (14). We previously noted an increased frequency of antigen-specific CXCR5^lo^Blimp1^+^ non-T_FH_ cells and a decreased CXCR5^+^ T_FH_ population upon immunization with KLH conjugated with lymphocytic choriomeningitis virus (LCMV) glycoprotein 61–80 peptide (KLH–gp_61_) in Alum+cGAMP compared to Alum alone on day 7 (15). To explore whether cGAMP elicits variant CD4 T cell responses to different antigens, mice were immunized with the KLH-gp_61_ antigen in Alum alone or Alum+cGAMP. Consistent with our previous findings (15), cGAMP substantially reduced the frequency of GC T_FH_ cells on day 8, which is considered the peak T_FH_ response (**Figure 3E** and **Supplementary Figure 2A**). Although the frequency of GC T_FH_ cells was lower in the Alum+cGAMP, the absolute number of GC T_FH_ cells was higher. Furthermore, B_PC_ differentiation post-KLH-gp61 immunization on day 8 was elevated compared to that with Alum alone (**Supplementary Figure 2B**). This indicates that cGAMP’s influence on B_PC_ generation may not solely rely on the T_FH_ to non-T_FH_ ratio but also on the number of T_FH_ cells present during this stage.

To determine whether the disparity in T_FH_ frequency in response to the KLH-gp_61_ antigen was time-dependent, we evaluated the T_FH_ response on day 15 post-immunization. In this experiment, we performed adoptive transfer of SMARTA CD4 T cells to examine both antigen-specific and endogenous polyclonal CD4 T cell responses (**Supplementary Figure 2C**). On day 15, the frequency of CD45.1^+^ gp-specific GC T_FH_ was comparable between the Alum+cGAMP and Alum alone groups in the KLH-gp_61_-immunized mice (**Figure 3F**; upper panel). Intriguingly, while Alum+cGAMP markedly increased the frequency of GC T_FH_ cells in response to KLH-NP on day 15 (**Figure 3C**), the frequency of GC T_FH_ in the polyclonal CD4 T cell population (CD45.1^-^) in KLH-gp_61_-immunized mice treated with Alum+cGAMP was similar to that in the Alum alone group (**Figure 3F**; lower panel). These findings suggest that different conjugations to carrier proteins may induce varied T-dependent immune responses. Although the frequency of GC T_FH_ was lower on day 8 or similar on day 15 in the Alum+cGAMP group, the total number of GC T_FH_ cells was significantly higher in Alum+cGAMP-treated mice than those given Alum alone (**Figures 3E, F**). These observations suggest that cGAMP may initially favor non-T_FH_ differentiation over T_FH_ differentiation, especially in response to the KLH-gp_61_ antigen, yet it appears to support sustained T_FH_ responses over time, influencing both the proportion and absolute number of T_FH_ cells.

### cGAMP alters antigen-specific T_FH_ and non-T_FH_ cell profiles following immunization

**Figure 4:**
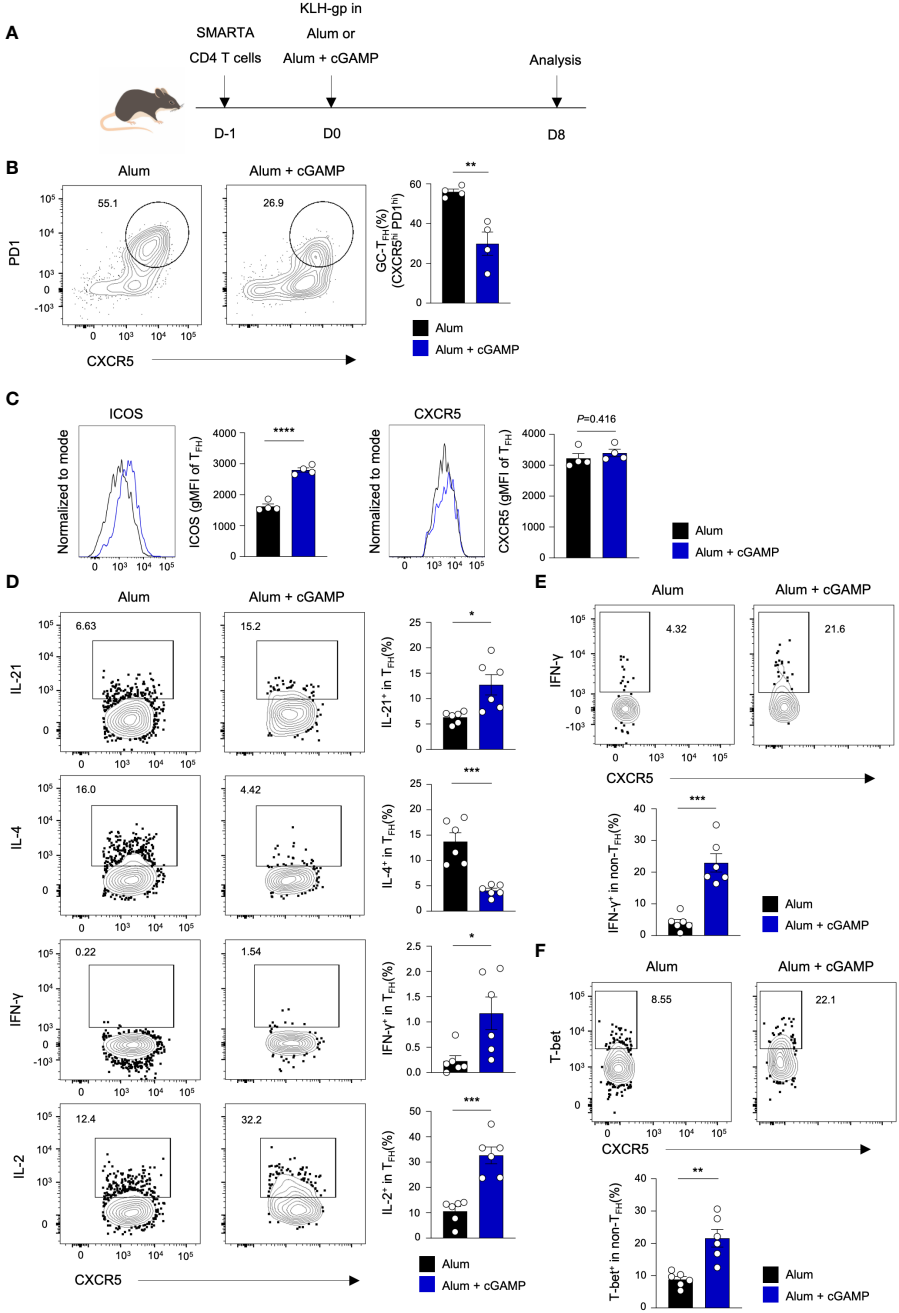
cGAMP alters antigen-specific T_FH_ and non-T_FH_ cell profiles following immunization. **(A)** SMARTA CD4^+^ T cells were adoptively transferred to mice followed by KLH-gp_61_ immunization in Alum alone or Alum+cGAMP, and analyzed after 8 days (n=4). **(B)** GC-T_FH_ populations were analyzed by flow cytometry. **(C)** ICOS and CXCR5 expression levels in the T_FH_ population are shown. **(D)** Flow cytometry analyses of gp_66_-restimulated IL-21^+^, IL-4^+^, IFN-γ^+^, and IL-2^+^ SMARTA cells in the T_FH_ population from KLH-gp_61_ immunized mice are shown (n=6). **(E)** IFN-γ^+^ SMARTA cells in the non-T_FH_ population are shown. **(F)** T-bet^+^ SMARTA cells in the non-T_FH_ population are shown. All experiments were performed twice, and each dot represents one mouse. Data are shown as mean ± SEM and were analyzed by using an unpaired two-tailed Student’s t-test. *p < 0.05, **p < 0.01, ***p < 0.001, ****p < 0.0001.

We further examined the characteristics of antigen-specific CD4 T cells on day 8 after immunization. The mice were adoptively transferred with SMARTA CD4 T cells, followed by immunization with KLH-gp_61_ in Alum alone or Alum+cGAMP (**Figure 4A**). Similar to the observation from polyclonal CD4 T cell responses on day 8, frequency of CXCR5^hi^PD1^hi^ GC T_FH_ cells decreased significantly in the Alum+cGAMP group compared to the Alum alone group (**Figure 4B**). Interestingly, the expression level of ICOS markedly increased in the Alum+cGAMP group, while those of CXCR5 was similar (**Figure 4C**). These suggest that although the frequency of GC T_FH_ was lower on day 8, the ICOS^+^ T_FH_ cells might have the potential for further differentiation into GC T_FH_ at a later time point. Moreover, Alum+cGAMP induced the production of IL-21, an important cytokine for the B_GC_ differentiation, in CXCR5^+^ T_FH_ cells (**Figure 4D**). Similarly, IL-2-producing T_FH_ cells also increased in the Alum+cGAMP group. In contrast, IL-4-producing T_FH_ cells were substantially decreased by cGAMP treatment, while the expression of IFN-γ was increased in T_FH_ population by cGAMP (**Figure 4D**). Furthermore, the majority population of CXCR5^low^ non-T_FH_ SMARTA cells was IFN-γ^+^ cells in mice treated with Alum+cGAMP, emphasizing that cGAMP enhanced T_H_1 response and repressed T_H_2 and T_FH_ responses at earlier immune response (**Figure 4E**). Correspondingly, the non-T_FH_ population expressed higher levels of the T-bet transcription factor in the Alum+cGAMP group (**Figure 4F**). Taken together, these results demonstrate that cGAMP downregulates GC T_FH_ differentiation in response to KLH-gp_61_ on day 8, but it improves its regulatory function over time.

## Discussion

Adjuvants are crucial for augmenting immunogenicity, particularly in protein subunit vaccines, significantly amplifying immune responses (16). Currently, various pathogen recognition receptor agonists are being developed to enhance the immunogenicity and efficacy of vaccines (17). The STING pathway, in particular, has been recognized for its role in both innate and adaptive immunity (9–11, 14, 18–21). cGAMP, a STING agonist, and its derivatives are being explored for use in conjunction with other adjuvants to combat a range of pathogens, including pan-sarbecoviruses and influenza (9, 10, 21). Our study adds the pivotal role of cGAMP in modulating the immune response, specifically by promoting the differentiation of B_GC_ and B_PC_. The enhancement of B_GC_ and B_PC_ populations, coupled with sustained antigen-specific antibody production following cGAMP administration, highlights the potential of the STING agonist as a potent vaccine adjuvant. The extended germinal center reaction and B_PC_ differentiation following cGAMP treatment indicate the establishment of a strong and lasting immune memory, a critical feature for sustained protective immunity.

In line with prior research on the effects of cGAMP on B cell activation (22), our results provide further insight into a type I Interferon-dependent mechanism by which cGAMP facilitates the differentiation of B_PC_. The reduction in B_PC_ frequency upon IFN-β blockade further confirms the importance of this pathway in immunoenhancing effects mediated by cGAMP. The involvement of IFN-β provides insights into the complex interaction between STING activation and the type I interferon pathway, essential for driving an effective B_PC_ immune response. The distinct response timings of B_PC_ and B_GC_ seem to be potentially influenced by IFN-β activity. Notably, the impact of cGAMP on B_PC_ peaked on day 8, after which it gradually diminished to levels comparable to the control group treated with Alum alone in response to the sRBD. This suggests that sustaining B_PC_ responses may necessitate extended innate signals. Indeed, boost immunization with cGAMP enhanced B_PC_ response on day 34 in response to KLH-NP. It also raises the possibility that the initial B_PC_ response could originate from extrafollicular B cells instead of B_GC_-derived cells. Furthermore, it is yet to be ascertained whether cGAMP’s influence on B cells, B_PC_, and CD4 T cells is attributable to direct intrinsic effects on each cell type or through indirect pathways. Addressing these questions will be an important focus for future studies.

Additionally, our study reveals a subtle yet significant influence of cGAMP on the differentiation of T_FH_, which is critical for GC reaction and the generation of high-affinity antibodies. The initial decline in GC T_FH_ cells on day 8 post-immunization with KLH-gp_61_, followed by later enhancement of T_FH_ function, offers a unique perspective on how cGAMP modulates the immune response over time. Although cGAMP appears to promote T_H_1 differentiation over T_FH_ responses initially, it may also facilitate sustained T_FH_ responses, likely advantageous for continuous antibody production. This biphasic effect indicates a sophisticated regulatory role of cGAMP, which could inform the optimization of vaccine approaches, particularly for diseases requiring a strong T_H_1 response.

In conclusion, our study not only corroborates the potent immunomodulatory properties of cGAMP but also highlights its complex, antigen-specific effects on T and B cell responses. These insights set the stage for subsequent studies into the mechanisms of cGAMP’s activity and its potential to enhance the efficacy of vaccines and precision immunotherapies.

## Data availability statement

The original contributions presented in the study are included in the article/**Supplementary Material**. Further inquiries can be directed to the corresponding author.

## Ethics statement

The animal study was approved by Institutional Animal Care and Use Committee of the College of Medicine at The Catholic University of Korea. The study was conducted in accordance with the local legislation and institutional requirements.

## Author contributions

MY: Data curation, Formal analysis, Investigation, Visualization, Writing – original draft, Writing – review & editing. YC: Investigation, Writing – review & editing. TW: Investigation, Writing – review & editing. YSC: Conceptualization, Resources, Writing – review & editing. JC: Conceptualization, Data curation, Formal analysis, Funding acquisition, Investigation, Methodology, Resources, Supervision, Validation, Visualization, Writing – original draft, Writing – review & editing.
