# Supplementary material for: The role of cGAMP via the STING pathway in modulating germinal center responses and CD4 T cell differentiation
Source: Front Immunol. 2024 Apr 12;15:1340001. doi: 10.3389/fimmu.2024.1340001 (PMC11045936; doi:10.3389/fimmu.2024.1340001)
Supplement: Supplementary file 1 [file DataSheet_1.pdf]

Supplementary Materials

Supplementary Figures

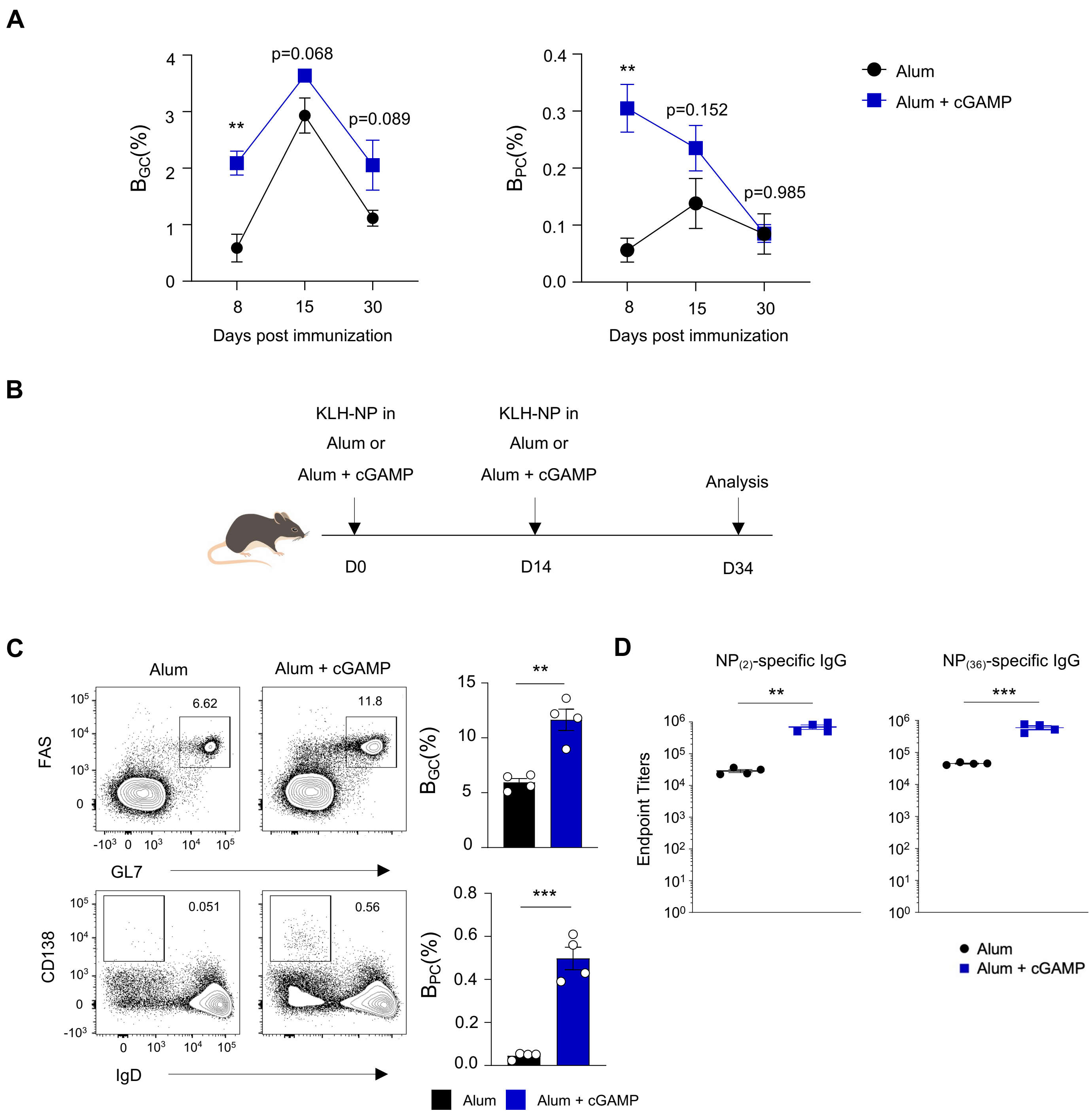

Supplementary Figure 1. B cell differentiation and antigen-specific antibody responses to sRBD and KLH-NP. (A) Frequencies of B_{GC} and B_{PC} in response to sRBD were measured at 8, 15, and 30 days post-immunization. (B) Mice underwent priming on day 0 and a booster on day 14 with KLH-NP in Alum or Alum+cGAMP. Draining LNs and blood were collected 20 days after the booster (day 34). (C) Flow cytometry plots show the percentages of B_{GC} ($GL7^{hi}FAS^{hi}$) and B_{PC} ($IgD^{lo}CD138^{hi}$) on day 34. (D) The high- and low-affinity NP-specific IgG endpoint titers were measured. **$p < 0.01$, ***$p < 0.001$.

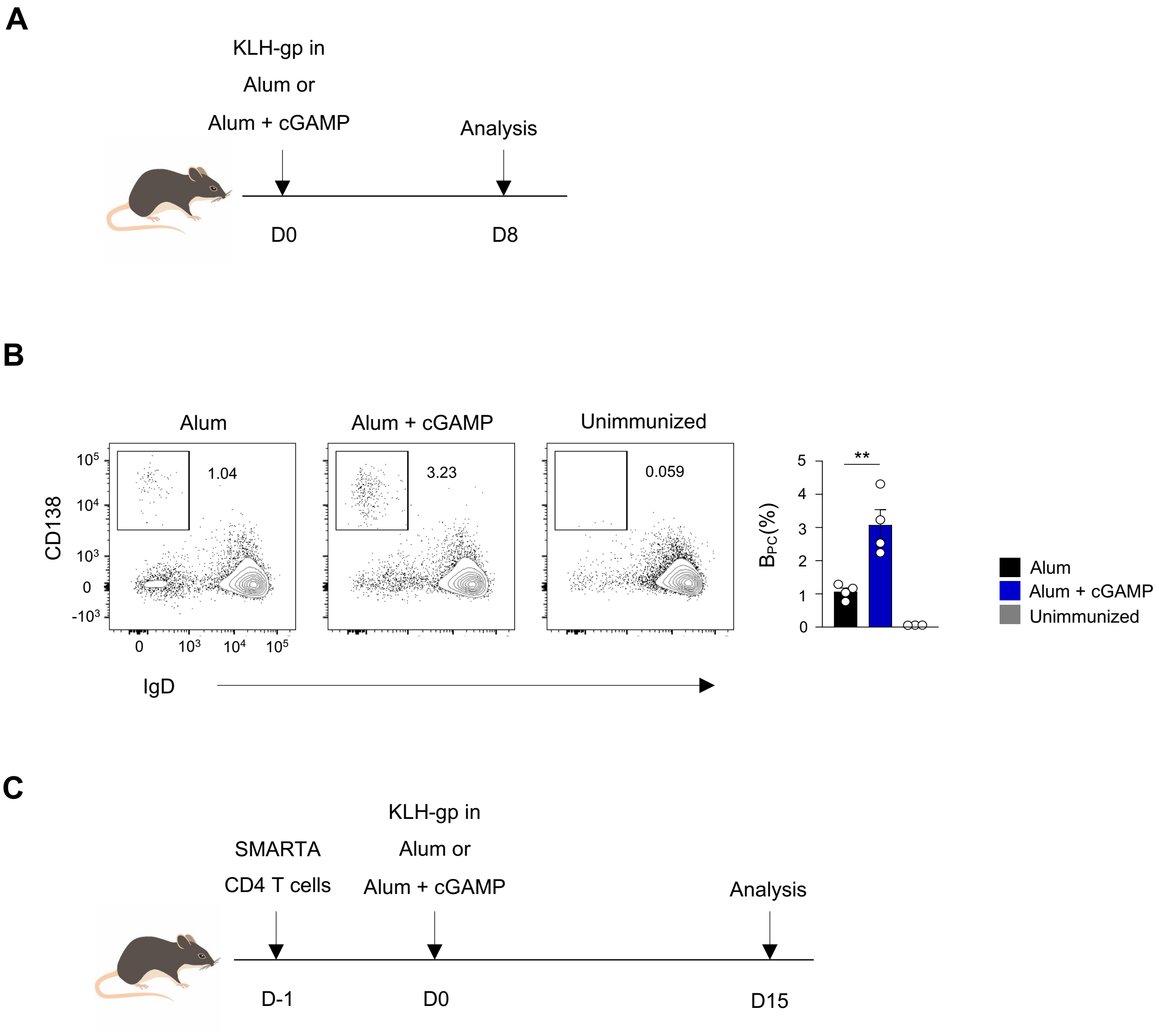

Supplementary Figure 2. B cells differentiation responses to KLH-gp$_{61}$. (A) Mice were immunized with KLH-gp$_{61}$ in Alum or Alum+cGAMP and analyzed at 8 days post-immunization. (B) Flow cytometry plots show the percentages of B$_{PC}$ on day 8. (C) SMARTA CD4^{+} T cells were adoptively transferred into mice, followed by KLH-gp$_{61}$ immunization in Alum or Alum+cGAMP and analyzed on day 15. **$p < 0.01$.
